# Supplementary material for: Collaborative intelligence in AI: Evaluating the performance of a council of AIs on the USMLE
Source: PLOS Digit Health. 2025 Oct 9;4(10):e0000787. doi: 10.1371/journal.pdig.0000787 (PMC12510544; doi:10.1371/journal.pdig.0000787)
Supplement: S2 File — (ZIP) [file pdig.0000787.s002.zip › S2 Data Raw data files containing unprocessed question inputs and LLM outputs including transcripts of deliberations/STEP3_Question-7-1_Type-MC-NJ.docx]

**Facilitator** **[Round 1]:** Researchers from a large neurology center are conducting a study to examine the relationship between stress levels and fibromyalgia-related pain. Two hundred patients with fibromyalgia are randomly selected from the center for participation in the study. During recruitment, a baseline interview is conducted with each participant to obtain sociodemographic and personality data. Participants are given a study-provided portable digital device and asked to maintain a daily schedule of their fibromyalgia pain and stress levels for 30 days using two well-validated and reliable self-reporting scales. Results show a positive and statistically significant relationship between stress levels and fibromyalgia-related pain (P<.05). Similarly, statistically significant relationships were found between Type A personality participants and increased stress levels (P<.01), as well as increased levels of fibromyalgia-related pain (P<.005). Which of the following is the most appropriate method to control for the potential confounding effect of patients with a Type A personality? (A) Matching (B) Post hoc analysis (C) Randomization (D) Standardization (E) Stratification

**AI_D:** RESTATING THE QUESTION: The study at hand is focused on analyzing the relationship between stress levels and pain related to fibromyalgia in two hundred patients selected randomly from a neurology center. Instruments capturing sociodemographic data and personality indicators were collected at baseline, and patients were asked to record their daily pain and stress levels using validated scales. Findings illustrated a statistically significant positive correlation between stress levels and fibromyalgia pain, with Type A personality patients showing higher stress and pain levels. The question is centered around identifying the best method to balance the confounding factor of Type A personality in this setting. PLANNING: The initial assumptions in this scenario are that the Type A personality of some participants could be a confounding variable that has affected the results of the study. Possible alternatives could be that Type A personality does not have a substantial confounding impact, and the observed effects are mostly due to other factors. Another could be that the Type A personality indeed influences the outcomes significantly. Clinically, the second assumption takes precedence due to the known characteristic of Type A individuals to be more stressed or tense, potentially intensifying symptoms of conditions like fibromyalgia. CLINICAL REASONING: The confounding effect of Type A personality could be controlled by various methods. Matching involves pairing participants based on the confounding variable (Type A personality) for comparison with others similar to them, but this may not be practical due to other varying factors. Post hoc analysis, a secondary analysis performed after the primary one, could help, but it will not prevent the confounding issue. Randomization, already implemented in participant selection, should ideally balance confounders across study groups but may not entirely remove the effect of potent confounders like Type A personality on the outcome. Similarly, standardization allows for comparison across different groups but doesn't resolve confounding. Stratification, on the other hand, involves dividing participants into groups based on the confounding variable (like Type A or not), treating these as separate experiments, which can help to control the confounding effect. CONCLUDING: Here, the most fitting method seems to be stratification. By dividing participants based on their Type A personality status, investigators can quantify and manage the varying effect of this personality type on stress levels and fibromyalgia pain within these separate strata. This method allows the scientists to understand the isolated impact of stress on fibromyalgia pain while adjusting for the personality type. RESPONSE: (E) Stratification is the most appropriate method to control for the potential confounding effect of patients with a Type A personality in this study.

**AI_A:** RESTATING THE QUESTION: To rephrase the question in simpler terms, it seeks the best approach to account for or negate the influence of having a Type A personality, which is a probable confounding variable, on the outcomes of a study comparing stress levels and fibromyalgia-related pain. Type A personalities are characterized by traits such as being highly competitive, ambitious, impatient, and often feeling a sense of urgency. A confounding variable is an extraneous attribute that can influence the results of a study by directly impacting both the dependent (fibromyalgia-related pain and stress levels) and independent variables (participant type). PLANNING: Let's dissect the potential corrective methods: (A) Matching attempts to associate participants within the study who have analogous characteristics to reduce the impact of confounding variables, but it may not be the most effective method because other uncontrolled or unrecognized confounding factors may still exist. (B) Post hoc analysis is a statistical method that is performed after data collection is complete. It might not be an efficient way to control confounding factors, as its goal is primarily to identify trends or correlations that were not initially hypothesized. (C) Randomization plays a distinct role in observational studies to minimize bias by placing participants into distinctive groups randomly, thereby promoting balance between the groups. (D) Standardization does allow for comparison, but it is also not a method to control for confounding variables. (E) Stratification divides subjects into subsets (strata) and analyzes them separately to reduce confounding. It allows for a more in-depth analysis of different subgroups in the research. CLINICAL REASONING: Type A personality is identified as a potential confounding factor in the relationship between fibromyalgia pain and stress levels. Managing these confounding factors is crucial for study outcomes' credibility. Given that the study is already underway and confounding by the 'Type A personality' trait is identified, stratification seems to be the clinically prioritized assumption for control. This method would involve dividing participants into strata (groups) based on their personality type and then analysing these strata separately. CONCLUDING: The goal here is to determine the accurate epidemiological association between stress levels and fibromyalgia-related pain irrespective of the impact of Type A personality. Each of the methods listed in the answer choices has its strengths and weaknesses, but given the context of this study and the identified confounding variable, stratification stands out as the most suitable choice. RESPONSE: Therefore, the most appropriate method to control for the potential confounding effect of patients with a Type A personality is (E) Stratification.

**AI_C:** RESTATING THE QUESTION: The query is asking for the most suitable method to handle the potential skewed effect of patients who have a 'Type A personality' in a research difficult to gauge the impact of stress levels on the pain associated with fibromyalgia. In other words, it is trying to identify the best approach to control the distortion that could arise from including patients with a Type A personality in the study given their propensity to experience higher stress levels and therefore elevated levels of fibromyalgia-related pain. PLANNING: The study's conclusion reveals a significant relationship among stress levels, Type A personalities, and fibromyalgia-related pain. This relationship might misrepresent the actual independent relationship between stress and fibromyalgia pain. A Type A personality might indeed act as a confounding factor that distorts this relationship. This potential confounding calls for a control strategy, such as matching, post hoc analysis, randomization, standardization, or stratification. CLINICAL REASONING: Let's examine each option: (A) 'Matching' refers to the pairing of subjects based on comparable confounding variables, which might not be practical in this case; (B) 'Post hoc analysis' involves statistical analyses that were not initially planned, and while it could possibly be applied, it might not be the most proactive solution; (C) 'Randomization' is used in the initial design of a study to equalize unknown or unmeasured confounders across groups, but in this case, the study has already been conducted; (D) 'Standardization' adjusts the confounding variable's distribution across groups to a common measure, which might still present some limitations; (E) 'Stratification' divides participants into distinct strata or levels based on the confounding variable, and it could be a sounder method to control for Type A personalities. CONCLUDING: Based on the aforementioned explanations and the study's design, 'stratification' seems to be the most appropriate method to control for the potential confounding effect of patients with a Type A personality in a study examining the correlation between stress levels and fibromyalgia-related pain. RESPONSE: The most suitable method to control for the potential confounding effect of patients with a Type A personality is Stratification (E).

**AI_E:** The question is asking about controlling a potential confounding effect in a study, particularly those arising from the presence of patients with a Type A personality. Type A personality is characterized by a constant sense of urgency, competitiveness, and hostility. This parameter is significant because the study revealed a connection between this personality type, elevated stress levels, and higher pain related to fibromyalgia, therefore acting as a potential confounding factor. The confounding factor refers to unrelated variables that correlate with both the dependent and the independent variable, potentially affecting the results of a study. The task here is to determine which approach—matching, post hoc analysis, randomization, standardization, or stratification—would be best congenial in controlling this confounding effect. Planning involves hypothesizing about each potential option for controlling the confounding effect. We’ll consider how each option works in relation to the prevalently scientific context. The matching refers to pairing participants with similar characteristics in the control and test groups to minimize data distortion. Post hoc analysis involves a detailed study of already collected data to understand the confounder's impact. Randomization, which involves randomly assigning subjects to test or control groups, can ensure that the confounder is evenly distributed. Standardization implies adjusting and organizing the data to control for confounding factors. Lastly, stratification divides participants into subgroups or strata based on the confounder. By clinical reasoning, while matching is effective in controlling for confounding by equating participants' characteristics, it may not be effective here unless a person with a matching personality type can be found for every study participant, which is challenging. Post hoc analysis, on the other hand, is useful for analyzing the influence of confounders after data collection but does not control the confounding effect during the study. Randomization is already applied in the study, but it fails to address the confounding effect of a Type A personality. Standardization, although used for controlling confounding factors, it may not be suitable here since variations in stress levels among individuals are dynamic and may not be standardized. Stratification seems the most suitable option. It involves separating the participants into strata or layers, i.e., Type A and non-Type A personalities in this case. This would allow researchers to analyze the effects of stress levels and fibromyalgia on two sets of data separately. This method controls for the confounding effect by treating it as an variable under study separately among the different strata. Therefore, using a step-by-step explanation that connects one sentence to the next with a clinical thought process, it is concluded that stratification (E) is the most appropriate method to control for the potential confounding effect of patients with a Type A personality in this study.

**AI_B:** The question is asking about the best method to control for a potential confounding effect of patients with a Type A personality in a study that is examining the relationship between stress levels and fibromyalgia-related pain. In the context of the study, a confounding variable is a factor other than the independent variable that might produce an effect in an experiment. In this case, the Type A personality is considered as a potential confounder because it shows significant relationships with both increased stress levels and increased levels of fibromyalgia-related pain. The strategies provided in the options (A to E) are different methods that are used in research studies to control for confounding variables. Before deciding which method to be clinically prioritized, each option should be questioned about its suitability in this case. Matching (A) involves pairing participants with similar characteristics, such as a Type A personality. Would that approach eliminate the confounder? Post hoc analysis (B), allows researchers to look at the data after the study is completed to see if other relationships are present, but doesn't necessarily control for the confounder. Randomization (C) often helps reduce the effect of confounders but in this study, patients have already been randomly selected so it might not be the best method in this case. Is standardization (D), which defines uniform procedures and criteria for the measurement of the variables, the best in this context? Stratification (E) involves dividing participants into subgroups based on the confounding variable. Would this approach allow control for the impact of the Type A personality? Based on my understanding, stratification should be given priority in this context. The advantage of this approach is that it can provide an explicit control for the confounding factor by assessing the effect of the primary exposure within strata of the confounding variable. To elaborate, we can consider each of the suggested methods for controlling confounding: Matching helps to ensure comparability between participants by only considering those that share similar characteristics, thereby controlling for these confounding variables. However, it may not necessarily eliminate the confounding variables. Post hoc analysis is performed after the main analysis and often considered as a deviation from the initial research plan. Therefore, using a post hoc analysis may not effectively control the confounding effect of Type A personality. Randomization, in which all participants have an equal chance of being assigned to either group, helps to balance known and unknown confounding variables. But in this study, where participants have already been randomly selected, the randomization process might not have been enough. Standardization introduces guidelines that ensure uniformity throughout the experiment. However, it predominantly focuses on the handling of data collection and does not directly deal with the control of confounding variables. Finally, stratification involves dividing participants into subgroups based on the confounding variable. In this context, participants could be stratified into Type A and non-Type A personality groups, and the effect of stress levels on fibromyalgia-related pain could be studied separately in these two strata. This would control the influence of Type A personality on the outcome. In conclusion, using stratification (E) to control for the potential confounding effect of patients with a Type A personality in this study seems to be the most appropriate method. This is because stratification directly handles confounding variables by enabling the confounding effect of a variable to be controlled in the analysis. Therefore, the inference drawn from the study becomes more reliable and valid.

**Facilitator** The question seeks to identify the most effective method to control for the potential confounding effect of patients with a Type A personality in a study examining the relationship between stress levels and fibromyalgia-related pain. Type A personality, characterized by traits such as competitiveness, ambition, impatience, and a sense of urgency, is identified as a potential confounding factor due to its significant relationships with both increased stress levels and fibromyalgia-related pain. The potential methods to control for this confounding effect include matching, post hoc analysis, randomization, standardization, and stratification. Matching involves pairing participants with similar characteristics, but it may not be practical due to other varying factors. Post hoc analysis, performed after the main analysis, could help identify other relationships but will not prevent the confounding issue. Randomization, which involves randomly assigning subjects to test or control groups, can ensure that the confounder is evenly distributed, but in this study, where participants have already been randomly selected, the randomization process might not have been enough. Standardization introduces guidelines that ensure uniformity throughout the experiment, but it predominantly focuses on the handling of data collection and does not directly deal with the control of confounding variables. Stratification, on the other hand, involves dividing participants into groups based on the confounding variable. In this context, participants could be stratified into Type A and non-Type A personality groups, and the effect of stress levels on fibromyalgia-related pain could be studied separately in these two strata. This would control the influence of Type A personality on the outcome. Therefore, based on the context of this study and the identified confounding variable, stratification stands out as the most suitable choice. It provides an explicit control for the confounding factor by assessing the effect of the primary exposure within strata of the confounding variable, thereby making the inference drawn from the study more reliable and valid. Hence, the most appropriate method to control for the potential confounding effect of patients with a Type A personality is Stratification (E).
